# Juvenile Neuromuscular Systems Show Amplified Disturbance to Muscle Unloading

**DOI:** 10.3389/fphys.2021.754052

**Published:** 2021-10-25

**Authors:** Michael R. Deschenes, Leah G. Patek, Audrey M. Trebelhorn, Madeline C. High, Rachel E. Flannery

**Affiliations:** ^1^Department of Kinesiology and Health Sciences, College of William & Mary, Williamsburg, VA, United States; ^2^Program in Neuroscience, College of William & Mary, Williamsburg, VA, United States

**Keywords:** disuse, neuromuscular junction, synapse, strength, fatigue, power 3

## Abstract

Muscle unloading results in severe disturbance in neuromuscular function. During juvenile stages of natural development, the neuromuscular system experiences a high degree of plasticity in function and structure. This study aimed to determine whether muscle unloading imposed during juvenile development would elicit more severe disruption in neuromuscular function than when imposed on fully developed, mature neuromuscular systems. Twenty juvenile (3 months old) and 20 mature (8 months old) rats were equally divided into unloaded and control groups yielding a total of four groups (*N* = 10/each). Following the 2 week intervention period, soleus muscles were surgically extracted and using an *ex vivo* muscle stimulation and recording system, were examined for neuromuscular function. The unloading protocol was found to have elicited significant (*P* ≤ 0.05) declines in whole muscle wet weight in both juvenile and mature muscles, but of a similar degree (*P* = 0.286). Results also showed that juvenile muscles displayed significantly greater decay in peak force due to unloading than mature muscles, such a finding was also made for specific tension or force/muscle mass. When examining neuromuscular efficiency, i.e., function of the neuromuscular junction, it again was noted that juvenile systems were more negatively affected by muscle unloading than mature systems. These results indicate that juvenile neuromuscular systems are more sensitive to the effects of unloading than mature ones, and that the primary locus of this developmental related difference is likely the neuromuscular junction as indicated by age-related differences in neuromuscular transmission efficiency.

## Introduction

The neuromuscular system is one of the critical integrative physiological organ systems featured in all animals, including humans (Silverthorn, 2019; American Physiological Society, 2021). In it, neural impulses generated by the motor nervous system are transmitted to skeletal muscle tissue resulting in muscle contraction. These contractions, in turn, are used to elicit movement of the body or its appendages. This movement can be used to complete physical work such as transporting objects, climbing stairs, home maintenance, as well as permit participation in recreational events including vigorous sports, gardening, and other enjoyable events. The neuromuscular system is also vital to maintaining health in that it helps regulate blood glucose levels, enables ventilation, strengthens bones of the skeletal system, while the physical exercise allowed by the neuromuscular system even counters psychological depression and sharpens mental function (Yue et al., 2017; Domingo-Horne and Salajegheh, 2018; Chiu et al., 2020).

For all of the benefits a healthy and optimally functioning neuromuscular system can provide, it must mature properly during the juvenile phase of life, particularly during the stages of rapid growth and development that occurs during early phases of adolescence (Sanes and Lichtman, 1999; Craig et al., 2016; DiStefano et al., 2017; Yasar et al., 2018). Indeed, it has been demonstrated that disturbances imposed on the neuromuscular system at this crucial juncture of physical maturation leads to severe muscle atrophy, and presumably, function (Gabel et al., 2017; Yue et al., 2017). The neuromuscular junction (NMJ), which is the vital synapse joining motor neurons to the skeletal myofibers they innervate, similarly experiences rapid rates of development during early years of adolescence (Hughes et al., 2006; Ferraro et al., 2012). This is important because disturbances in synaptic activity of the NMJ disrupts normal function of skeletal muscle and its capacity to perform the necessities, and rigors of daily living (Metter et al., 2002; Newman et al., 2006; Hairi et al., 2010). And while subtotal disuse of the neuromuscular system, i.e., unloading, immobilization, bed rest, has clearly been shown to disrupt neuromuscular function in adults (Booth and Gollnick, 1983; Hackney and Ploutz-Snyder, 2012; Monti et al., 2021), little is known about how rapidly growing, maturing neuromuscular systems react to the imposition of disuse. Thus, the objective of the present investigation was to compare the deleterious effects of disuse in the form of muscle unloading on mature, fully developed vs. juvenile, rapidly developing neuromuscular systems characterized by accelerated plasticity.

## Materials and Methods

### Subjects and Treatment

Twenty young adult (8 months), and 20 juvenile (3 months) male Wistar rats were purchased from Charles River Laboratories (Wilmington, MA, United States). At these ages, rats would be the approximate equivalent of 24 and 9 years old in human years (Sengupta, 2013). For the present project, these age categories were referred to as Mature and Juvenile, respectively. At 3 months of age, rats were undergoing a period of rapid accumulation of muscle mass (Turturro et al., 1999; Quinn, 2005). Within each age category, half of the animals were randomly assigned to either muscle unloading (UL), or control (CTL) conditions for the 2 week intervention period, yielding a total of four treatment groups, i.e., Mature-UL, Mature-CTL, Juvenile-UL, and Juvenile-CTL. In all cases, muscle unloading was imparted with the hindlimb suspension model first described by Morey et al. (1979). In this model, hindlimb muscles are unloaded by preventing the animal’s weight-bearing and locomotor activities by elevating its back feet just enough to eliminate contact with the floor. This is achieved by securing adhesive strips along two sides – lengthwise – of the subject’s tail and attaching a clip to the strips which is then secured to a swivel device suspended above the animal. This enables the rat to move about in a full 360° arc using its forelimbs. Hindlimb suspended animals remained in this condition 24 h/day for 14 consecutive days. All unloaded animals were provided with standard rat chow pellets ground to smaller fragments with an ice crusher making food easier to handle, thus promoting eating to avoid excessive weight loss while unloaded. All animals were provided with both food and water *ad libitum* during the experimental period. Wood shavings lined the floor beneath, and environmental conditions featured an ambient air temperature of 21–22°C with a relative air humidity of ∼50%.

Animals assigned to control conditions were housed individually in plastic tubs lined with wood shavings, with water and food provided *ad libitum*. These Control animals were allowed to move about their tubs at will promoting normal weight-bearing and locomotor activities. All treatment and care procedures employed in this investigation were approved by the institution’s animal care and use committee which operates in full compliance with the National Institutes of Health Guide for the Care and Use of Laboratory Animals as revised in 2011.

### *Ex vivo* Muscle Stimulation/Recording Procedure

Upon the conclusion of the 2 week intervention period, neuromuscular function of the soleus muscle of all animals was assessed with an *ex vivo* stimulation and recording system (system 1205A with ASI 615A data acquisition and analysis system with LabView Windows software, Aurora Scientific Inc., Aurora, ON, Canada). The soleus was selected for study as it is the primary weight bearing muscle, and it is heavily recruited during locomotor activity in the rat (Laughlin and Armstrong, 1985; Roy et al., 1991), and accordingly, was likely to be substantially affected by hindlimb suspension. Upon anesthesia administered with a ketamine/xylazine cocktail of 50/10 mg/kg of body mass, soleus muscles were surgically removed and for 15 min incubated in Ringer Solution (137 mM NaCl, 4.7 mM KCl, 3.4 mM CaCl_2_, 1.2 mM MgSO_4_, 1 mM NaH_2_PO_4_, 112 D-glucose, pH = 7.4) that was vigorously aerated with a gas combination of oxygen (95%) and carbon dioxide (5%) and maintained at 21–22°C. Following this incubation, the muscle was subjected to an electrical stimulation protocol that first established optimal muscle length to determine peak isometric force. With the use of platinum plate electrodes, the stimulation parameters used mimicked those employed earlier (Lomo and Rosenthal, 1972) which alternated indirect (neural) and direct (sarcolemma) stimulation so that neuromuscular transmission efficiency could be quantified. The stimulation protocol consisted of a series of sets featuring nine pulses at a constant 37 V and ∼25 Hz for a duration of 0.2 ms (indirect) followed by a single 2 ms pulse (direct) so that the duration of each set was 30 s, with a total of 10 sets comprising the 5 min stimulation protocol. During longer pulses, myofibers are stimulated directly via the sarcolemma, but also due to their greater sensitivity, nerve terminal endings on the myofibers are stimulated as well, but are not responsible force the muscle fore produced (Mines, 1913; Bowman et al., 1962; Bowman, 2006). Further investigations using NMJ blockers (i.e., d-tubocurarine) were conducted to verify that the longer duration of pulses do, in fact, directly stimulate myofibers (Hultman et al., 1983; Aldrich et al., 1986; Kuei et al., 1990). It is only during the shorter pulses (0.2 ms) intended to exclusively stimulate nerve terminal endings that the NMJ is vital to controlling muscle function as the longer stimuli (2 ms) directly activate the sarcolemma, thus bypassing the role normally played by the NMJ (Pagala et al., 1984; Deschenes et al., 2016; Fogarty et al., 2020). Sets were repeated continuously for a total duration of 5 min for the protocol with data being collected at the beginning and end of each of the 10 sets of the protocol.

### Neuromuscular Performance Measures Assessed

Peak muscle force produced while being stimulated both directly, and indirectly was quantified, as was specific tension, or peak force produced relative to whole muscle wet weight. Moreover, neuromuscular power, or time needed to reach peak force production, was evaluated during the beginning and end of the 5 min train of stimuli. The fatigability of the neuromuscular system was determined by measuring the loss of contractile force generated during the course of the 5 min stimulation protocol, whether produced directly or indirectly.

Particularly germane to this study was assessing efficiency of the NMJ in transmitting the pre-synaptic electrical signal arriving at the synapse to the post-synaptic sarcolemma resulting in muscle force production. This is functionally derived by determining the difference in contractile force produced via indirect stimulation relative to the force generated directly. The closer these two figures are to each other, the more impressive the neuromuscular transmission efficiency, and thus NMJ function. Neuromuscular efficiency was quantified as indirect maximal force divided by direct maximal force, multiplied by 100.

### Statistical Analysis

All results are displayed as means ± SE. The percent decline in muscle mass over the 2 week intervention among Mature and Juvenile solei was compared with unpaired *t*-tests. All other variables of interest were assessed with 2-way ANOVA with main effects for age (Mature vs. Juvenile), and treatment (Control vs. Unloaded) as well as interaction between those main effects, under each type of stimulation (indirect vs. direct) of stimulation. When indicated, i.e., significant interaction, a Tukey *post hoc* analysis was conducted to identify significant pairwise differences. In all analyses, *P* ≤ 0.05 was used to establish significance.

## Results

### Body Mass and Muscle Weight

When examining the effects of maturity and unloading on body mass and soleus muscle wet weight, a main effect of age was noted before the intervention period with Mature rats weighing more than Juvenile ones (*P* < 0.0001). There was, however, no effect for treatment (*P* = 0.595) with CTL and UL rats displaying similar body weights, nor was there a significant interaction between age and treatment (*P* = 0.648). Following the 2 week intervention period, a significant (*P* < 0.0001) main effect of age persisted with Mature rats still weighing more than Juvenile ones. It was also determined that at post-intervention, treatment assignment (CTL vs. UL) had a significant (*P* = 0.0001) impact on body mass, as UL animals displayed less body mass than CTL rats. Moreover, a significant (*P* = 0.019) interactive effect was detected following the 14 day intervention. *Post hoc* analysis revealed that except for Juvenile-CTL vs. Juvenile-UL, all pairwise differences were found to be significant.

Soleus muscle wet weights could be compared only after the intervention period when animals were euthanized. In this variable, *t*-test results comparing relative (%) loss of soleus wet weight between Mature and Juvenile muscles revealed that the degree of muscle atrophy due to unloading was similar for adult and young rats. When examining ANOVA results from muscle wet weight analysis, significant effects for age (*P* < 0.0001, Mature > Juvenile), treatment (*P* < 0.0001, CTL > UL), and interaction (*P* = 0.050) were identified. *Post hoc* analyses revealed that with the exception of Juvenile-CTL vs. Mature-UL, all pairwise differences were significant (all *P* < 0.002). ANOVA results concerning body mass and muscle wet weight are found in Table 1.

**TABLE 1:** Body mass (g) before (Pre) and after (Post) the 2 week unloading intervention and soleus whole muscle wet weight (mg) after that intervention.

	Pre, Body mass*	Post, Body mass*^†⁣‡^	Soleus wet weight*^†⁣‡^
Juvenile CTL	161.9 ± 3.7	244.1 ± 6.6	119.3 ± 4.9
Juvenile HS	162.1 ± 3.0	233.1 ± 4.8	83.5 ± 6.7^#^
Mature CTL	358.7 ± 3.4	382.1 ± 5.8^#^	186.1 ± 8.9^#^
Mature UL	362.3 ± 4.2	340.6 ± 7.^#^	119.6 ± 9.5

*Values are mean ± SE, *N* = 10/group.*

*Indicates significant (*P* ≤ 0.05) main effect for age (Mature > Juvenile).

*^†^Indicates significant (*P* ≤ 0.05) main effect for treatment (CTL > UL).*

*^‡^Indicates significant (*P* ≤ 0.05) interaction between age and treatment.*

*^#^Indicates significant (*P* ≤ 0.05) difference from all other groups.*

*CTL, control; UL, unloaded.*

### Peak Muscle Force

In our stimulation protocol, we used a stimulation frequency (25 Hz) that is in the physiological range (Eken, 1998; Mildren et al., 2019), but less than that typically used during testing exclusively for maximal force production which does not feature prolonged stimulation bouts as employed here. Still, it is reasonable to make between group, and between comparisons time intervals as done here. With this physiological stimulation frequency it was determined that following the unloading period, it was established that during indirect, or neural, stimulation of muscles, there were significant effects for both age (*P* = 0.0002, Mature > Juvenile) and treatment (*P* = 0.0009, CTL > UL) with no evidence of interaction (*P* = 0.554). When examining results from direct, or sarcolemmal, stimulation, ANOVA findings were similar to those observed during indirect stimulation. That is, Mature solei again generated more force than Juvenile ones (*P* = 0.0001), while muscles from CTL rats were stronger than those of UL animals (*P* = 0.0001); there was no evidence of interaction (*P* = 0.455).

Results from measuring peak force during the last 30 s of the 5 min stimulation protocol during indirect (neural) stimulation revealed a significant (*P* = 0.031) main effect for age (Mature > Juvenile), but not for treatment (*P* = 0.102). Once again, no interactive effect between age and treatment was noted (*P* = 0.576).

When quantifying strength produced during direct (sarcolemmal) stimulation during the final set of the test protocol, a significant (*P* = 0.028) effect for age was highlighted (Mature > Juvenile). Also, a significant (*P* = 0.0004) effect for treatment was revealed showing that muscles that had been unloaded for 14 days were weaker than those remaining under weight-bearing control conditions. As with indirect stimulation procedures, no significant (*P* = 0.261) interaction between age and treatment was discovered during direct muscle stimulation. Data regarding development of peak force are presented in Table 2.

**TABLE 2:** Effects of age (mature/juvenile) and treatment (control/unloaded) on peak tension (*N*) at beginning (Initial) and conclusion (Final) of 5 min train of indirect (nerve) or direct (muscle) stimulation.

	**Initial**		**Final**	
	Indirect stimulation*^†^	Direct stimulation*^†^	Indirect stimulation*	Direct stimulation*^†^
Juvenile, CTL	25.4 ± 4.5	33.2 ± 4.0	8.7 ± 1.7	19.1 ± 2.2
Juvenile, UL	5.1 ± 3.0	7.9 ± 2.4	4.6 ± 1.3	6.5 ± 1.4
Mature, CTL	42.6 ± 6.5	50.3 ± 5.9	12.2 ± 2.5	22.0 ± 2.9
Mature, UL	28.1 ± 4.9	31.8 ± 4.9	9.2 ± 2.5	15.1 ± 3.0

*Values are mean ± SE, *N* = 10/group.*

*Indicates significant (*P* ≤ 0.05) main effect for age (Mature > Juvenile).

*^†^Indicates significant (*P* ≤ 0.05) main effect for treatment (CTL > UL).*

*CTL, control; UL, unloaded.*

### Specific Tension

When investigating specific tension, or force produced relative to muscle mass, by these same muscles, indirect stimulation during the first set of the test protocol indicated a significant effect for age (*P* = 0.011, Mature > Juvenile), along with treatment (*P* = 0.032, CTL > UL). Moreover, there was a significant (*P* = 0.046) interaction among main effects of age and treatment. *Post hoc* analysis for interaction showed that Juvenile-UL muscles exhibited significantly lower specific tension than the three other treatment groups when indirectly stimulated.

When specific tension was quantified during the first set using direct stimulation of the sarcolemma, results were similar to those revealed during indirect stimulation at the initial set of the testing procedure. That is, significant main effects for age (*P* = 0.012, Mature > Juvenile), treatment (*P* = 0.002, CTL > UL), and interaction (*P* = 0.008) were noted. Again, *post hoc* analysis for the interactive effect showed that juvenile muscles subjected to hindlimb suspension displayed significantly less specific tension than muscles form each of the other three treatment groups.

When comparing specific tension during the final set of the 5 in stimulation protocol, it was found that during indirect, or neural, activation there were neither main effects, nor interactive ones to be revealed. Yet when examining specific tension with direct stimulation during the same last set of performance, there was a significant main effect of treatment (*P* = 0.050, CTL > UL), and interaction between age and treatment (*P* = 0.038) with *post hoc* analysis detecting only one pairwise difference (Juvenile-CTL > Juvenile-UL). Specific tension findings are located in Table 3.

**TABLE 3:** Effects of age (mature/juvenile) and treatment (control/unloaded) on specific tension at beginning (Initial) and conclusion (Final) of 5 min train of indirect (nerve) or direct (muscle) stimulation.

	**Initial**		**Final**	
	Indirect stimulation*^†⁣‡^	Direct stimulation*^†⁣‡^	Indirect stimulation*^†⁣‡^	**Direct stimulation^†⁣‡^**
Juvenile, CTL	21.1 ± 3.7	27.9 ± 3.1	7.3 ± 1.5	15.9 ± 1.8^¶^
Juvenile, UL	6.2 ± 3.5^#^	9.5 ± 2.5^#^	4.0 ± 1.5	8.4 ± 1.4
Mature, CTL	23.2 ± 3.3	27.4 ± 3.3	6.5 ± 1.3	12.1 ± 1.8
Mature, UL	22.6 ± 3.4	26.1 ± 3.1	7.4 ± 1.7	12.3 ± 2.1

*Values are mean ± SE, *N* = 10/group. Specific tension calculated as peak tension in Newtons divided by whole muscle wet weight × 100, i.e., *N*/mg × 100.*

*Indicates significant (*P* ≤ 0.05) main effect for age (Mature > Juvenile).

*^†^Indicates significant (*P* ≤ 0.05) main effect for treatment (CTL > UL).*

*^‡^Indicates significant (*P* ≤ 0.05) interactive effect between age and treatment.*

*^#^Indicates significant (*P* ≤ 0.05) difference from all other groups.*

*^¶^ Indicates significant (*P* ≤ 0.05) difference from Juvenile, UL.*

*CTL, control; UL, unloaded.*

### Time to Peak Force

Because of its important role in defining overall neuromuscular function, time to peak force, was also evaluated with *ex vivo* stimulation. When using indirect, neural stimulation of isolated solei, during the first set of activation, a significant (*P* = 0.031) main effect for treatment was noted whereby unloaded muscles were found to develop peak force more quickly than control muscles. No significance was established for the main effect of age, or interaction between age and treatment. Yet when directly stimulating soleus muscle myofibers, different results were detected. That is, no significance was found for the effect of treatment, or interaction, but a trend (*P* = 0.082) was observed for a main effect of age whereby Mature muscles reached peak force more quickly than Juvenile muscles.

Statistical analysis disclosed that during the last set of the 5 min train of stimuli, no significant main effects, or interaction between age and treatment were identified. This was true whether the muscle stimulated via nerve terminal ending (indirect) or the sarcolemma (direct) of myofibers. Data regarding time to peak tension are found in Table 4.

**TABLE 4:** Effects of age (mature/juvenile) and treatment (control/unloaded) on time to peak tension (seconds) at beginning (Initial) and conclusion (Final) of 5 min train of indirect (nerve) or direct (muscle) stimulation.

	**Initial**		**Final**	
	**Indirect stimulation^†^**	**Direct stimulation**	**Indirect stimulation**	**Direct stimulation**
Juvenile, CTL	0.845 ± 0.035	0.916 ± 0.093	0.835 ± 0.030	0.877 ± 0.022
Juvenile, UL	0.645 ± 0.111	1.004 ± 0.068	0.855 ± 0.060	0.955 ± 0.040
Mature, CTL	0.748 ± 0.038	0.837 ± 0.080	0.919 ± 0.030	0.976 ± 0.030
Mature, UL	0.613 ± 0.090	0.810 ± 0.054	0.911 ± 0.042	0.869 ± 0.087

*Values are mean ± SE, *N* = 10/group.*

*^†^Indicates significant (*P* ≤ 0.05) main effect for treatment (CTL > UL), i.e., longer time to peak force.*

*CTL, control; UL, unloaded.*

### Neuromuscular Fatigue

Fatigue, or the percent decline in peak force during the 5 min train of stimuli, was also examined as an important parameter of neuromuscular function. When assessed during indirect stimulation, there were main effects for both age (*P* = 0.0003), and treatment (*P* = 0.0004), as well as interaction between those two factors (*P* = 0.0006). More specifically, it was found that Mature muscles exhibited a greater decrement in force during the train of stimuli, than did Juvenile muscles, and that Controls showed a greater loss of force than did Unloaded muscles (Recall that Mature and Control muscles had also demonstrated greater peak force). Importantly, *post hoc* analysis of the significant interactive effect indicated that the loss of contractile force during the 5 min train of stimulation was less in Juvenile-UL muscles than it was in each of the other three groups (Recall also, that these muscles had exhibited less force than the other groups initially). Similar results were found when contractile force of soleus muscles was elicited with direct (sarcolemma) stimulation. Again, main effects for age (*P* < 0.0001, Mature > Juvenile), and treatment (*P* = 0.0009, CTL > UL), with significant interaction (*P* = 0.010) were noted. And as with indirect stimulation, *post hoc* results indicated that Juvenile-UL muscles showed significantly less fatigue over the 5 min testing regimen than all other groups. All results concerning neuromuscular fatigue can be viewed in Table 5.

**TABLE 5:** Effects of age (mature/juvenile) and treatment (control/unloaded) on fatigue (% decline), during a 5 min train of indirect (nerve) or direct (muscle) stimulation.

	**Indirect stimulation** * ^† ‡^	Direct stimulation* ^† ‡^
Juvenile, CTL	67.1 ± 4.4	42.2 ± 3.0
Juvenile, UL	15.3 ± 10.2^#^	41.6 ± 7.7
Mature, CTL	82.7 ± 7.2	52.7 ± 5.7
Mature, UL	84.8 ± 3.7	58.1 ± 7.2

*Values are mean ± SE, *N* = 10/group. Fatigue calculated as percent decline in peak tension during 5 min train of stimulation.*

*Indicates significant (*P* ≤ 0.05) main effect for age (Mature > Juvenile).

*^†^Indicates significant (*P* ≤ 0.05) main effect for treatment (CTL > UL).*

*^‡^Indicates significant (*P* ≤ 0.05) interactive effect between age and treatment, whereby Juvenile, HS is less than all other groups during that interval.*

*^#^Indicates significant (*P* ≤ 0.05) difference from all other groups.*

*CTL, control; UL, unloaded.*

### Neuromuscular Transmission Efficiency

In the last neuromuscular variable of interest tested, neuromuscular transmission efficiency, or function of the NMJ, it was discovered that during the first set of the train of testing stimuli, there was a significant main effect for age (*P* = 0.001, Mature > Juvenile), but not for treatment. In examining the interaction between age and treatment, a significant (*P* = 0.019) effect was detected whereby synaptic function of Juvenile-UL systems was impeded significantly more than all other groups.

By the last set of the 5 min stimulation regimen, however, changes in neuromuscular transmission efficacy were evident. There was still a significant main effect for age (*P* = 0.035, Mature > Juvenile), and an absence of a significant effect for treatment, but at this juncture no evidence of significant interaction between age, and treatment (*P* = 0.749) could be detected. Findings regarding neuromuscular transmission efficiency are presented in Table 6.

**TABLE 6:** Effects of age (mature/juvenile) and treatment (control/unloaded) on neuromuscular transmission efficiency (%), at beginning (Initial) and conclusion (Final) of 5 min train of stimulation.

	Initial*^‡^	Final*
Juvenile, CTL	71.0 ± 6.3	40.6 ± 5.6
Juvenile, UL	42.9 ± 6.8^#^	41.6 ± 7.7
Mature, CTL	82.7 ± 7.2	52.7 ± 5.7
Mature, UL	84.8 ± 3.7	58.1 ± 7.2

*Values are mean ± SE, *N* = 10/group. Neuromuscular efficiency calculated as indirect peak tension/direct peak tension × 100.*

*Indicates significant (*P* ≤ 0.05) main effect for age (Mature > Juvenile).

*^‡^Indicates significant (*P* ≤ 0.05) interactive effect between age and treatment, whereby juvenile-HS is less than all other treatment groups.*

*^#^Indicates significantly (*P* ≤ 0.05) less than three other treatment groups.*

*CTL, control; UL, unloaded.*

## Discussion

The neuromuscular system is critical to an individual’s health and quality of life. As with other physiological systems, it has been shown to be capable of an impressive degree of plasticity. This is true in response to changes in its level of activity – evidence has established that both increased and decreased activity alter neuromuscular machinations and elicit physiological and morphological adaptations (Alshuaib and Fahim, 1990; Fahim, 1993; Valdez et al., 2010; Deschenes et al., 2016, 2020). This system, which features both motor neurons and the myofibers they innervate, may experience an expansion (high intensity, short duration) exercise (Volek et al., 1999; Stock et al., 2017; Lopez et al., 2021) or reduction (low intensity, long duration) exercise (Deschenes et al., 1993, 2018) in myofiber size. Decreased activity, however, invariably results in myofiber atrophy. This of course, is associated with decreased muscle force production upon contraction. Moreover, decreased activity has been shown to impede the nervous system’s capacity to recruit high threshold motor units responsible for high force generation and power, which is also described as explosive strength (Kraemer et al., 2002; Daly, 2017; Bahat et al., 2021). The increased strength accompanying high intensity resistance training, i.e., weight lifting, is attributed to both increased mass of contractile proteins, as well as greater neural drive to the contracting muscle tissue (Walker and Hakkinen, 2014; Ahtiainen et al., 2016).

In addition to the neuromuscular plasticity observed in response to alterations in activity, similar plasticity in neuromuscular function and structure naturally occurs throughout the lifespan beginning with, and even preceding, birth, all the way through the latest stages of senescence (Piasecki et al., 2016; Dobrowolny et al., 2021; Wakabayashi, 2021). There is a rich body of literature describing neuromuscular adaptations to aging which includes myofiber atrophy, loss of skeletal muscle mass, decline in number of motor neurons, and even thinning of the myelination of those remaining axons (Banker et al., 1983; Lexell et al., 1988; Deschenes, 2011; Sims-Robinson et al., 2013; Rudolf et al., 2014; Audouard et al., 2015; Willadt et al., 2018; Pratt et al., 2021).

Age-related disruptions in the structure and function of the NMJ, which enables communication between the nervous and skeletal muscle systems, have also been described (Badawi and Nishimune, 2018; Dobrowolny et al., 2021; Fuertes-Alvarez and Izeta, 2021; Pratt et al., 2021). Age-related plasticity of the NMJ include fragmentation of the post-synaptic endplate, as well as increased length and branch points of pre-synaptic nerve terminal endings (Deschenes et al., 2016, 2018; Bao et al., 2020), with greater dispersion, and a smaller number of acetylcholine (ACh) containing pre-synaptic vesicles (Petralia et al., 2014; Ivannikov and Van Remmen, 2015; Badawi and Nishimune, 2018). Numerous reports have also indicated that compared to young ones, aged neuromuscular systems are more vulnerable to the detrimental effects of disuse (Robbins, 1992; Pandya and Patani, 2021), suggesting that at times of enhanced natural neuromuscular plasticity, imposed neuromuscular disuse elicits more pronounced morphological and physiological remodeling than during the mature, adult phase of life that comprises much of the lifespan (Fahim, 1989; Deschenes and Wilson, 2003; Wilson and Deschenes, 2005).

That being said, it is also true that far less is known regarding the effects of reduced neuromuscular activity during another crucial phase of heightened plasticity, i.e., development. During this natural development, the neuromuscular system exhibits a wide range of remodeling and at an accelerated rate while striving to achieve its optimal level of functioning, i.e., young adulthood. And while such natural neuromuscular plasticity is well known (Lichtman, 1987; Schuetze and Role, 1987; Fernandes and Keshishian, 1999; Hoch, 1999; Epperson and Sandage, 2019), a real dearth of information exists regarding how altered activity influences the natural malleability demonstrated during that period of development in juvenile neuromuscular systems. In the present project, we aimed to shed light on how an intervention of disuse, i.e., muscle unloading, would modify neuromuscular function as revealed by a panel of physiological challenges. We did this by comparing unloading-induced disturbances in neuromuscular function in juvenile systems, with disruption in neuromuscular function evident in young adult systems when those two groups were similarly subjected to disuse.

The thinking going into this study was that because the neuromuscular systems of juvenile animals are already in a state of high plasticity as they are going through a period of rapid growth and development, they would be more susceptible to the deleterious effects of disuse. Indeed, our results confirmed our expectation. In fact, this was best supported by our finding that the loss of peak force generation caused by unloading in juvenile rats was of a magnitude that was more than twice that observed in mature animals. This was the case whether muscles were stimulated indirectly (79 vs. 34% loss), or directly (76 vs. 36% loss). Interestingly, the severe unloading-induced decrement in maximal force production was roughly the same whether muscle contraction was elicited by stimulating nerve terminal endings, or the sarcolemma. This made it difficult initially to attribute loss of strength specifically to either neural or myofiber impairment; both have been shown to negatively impact contractile force (Deschenes et al., 2017; Monti et al., 2021; Padilla et al., 2021). With little doubt, the difference in loss of peak force following unloading cannot be explained by differential adaptations in muscle size as results showed that adult and juvenile muscles experienced similar atrophy with unloading. This ambiguity with respect to the source of age-specific sensitivity to unloading led us to examine specific muscle tension – also known as muscle quality – to gain better insight as to the root cause of unloading-induced strength decline, and differences with maturity. Our data revealed that both when fresh, and when fatigued by the testing protocol, juvenile rats were far more affected by the preceding muscle unloading intervention. For example, when assessed at the start of the testing regimen, specific tension had been decreased 70% in juvenile muscles by unloading, while mature rats demonstrated a negligible (<1%) unloading-related difference. Similar results were noted whether isolated solei were stimulated directly, or indirectly via nerve terminal endings. These findings suggest that maturity had a powerfully mitigating effect on response to disuse when assessing muscle quality. They also begin to suggest that the neural system might be the primary locus of the severe unloading-induced impairment of muscle function in juvenile animals as specific tension produced by skeletal muscle is strongly influenced by performance of the NMJs of myofibers involved (Berg et al., 1997; Deschenes et al., 2008, 2016).

Our results related to fatigability during the 5 min testing protocol can easily be misconstrued at first glance, as it appears that at least in juvenile muscles, unloading attenuates the loss of force production during the testing protocol appearing as improved muscle endurance. This was evident whether an indirect, or direct muscle stimulation procedure was employed. This, however, is misleading in that the diminished fatigability, or decline in force production over 5 min, is mainly due to the fact that relative to all other experimental groups, juvenile unloaded rats were already markedly weakened, and thus had little potential for further decline in strength during testing. Previously, it has been shown that muscles with small initial amounts of strength display less fatigue during an endurance test than those with higher levels of strength (Hunter et al., 2006; Kent-Braun, 2009; Ha et al., 2021).

In terms of identifying the source(s) of the greater sensitivity of juvenile muscles to the negative consequences of neuromuscular disuse, perhaps the most insightful findings come from our measure of neuromuscular transmission efficiency. This measure quantifies the extent to which strength decline can be ascribed to impairment in the function of the NMJs of myofibers. In fresh, unfatigued muscle, our findings indicate that in mature muscle the NMJ faithfully transmitted neural signals to the myofibers as neuromuscular efficiency, or force produced with neural vs. muscle stimulation, did not differ between Control and Unloaded groups (both ∼ 85%). However, in juvenile muscles, hindlimb suspension interfered with proper synaptic function of the NMJ by ∼40% compared to the <3% difference observed in mature muscles. It was also noted that NMJ function was significantly lower in Juvenile-Unloaded, than in each of the other three treatment groups. However, by the end of the 5 min testing period, when the muscles showed signs of fatigue, neuromuscular transmission efficiency remained lower in juvenile muscles relative to mature ones, but unloading no longer affected juvenile and mature muscles differently. Combined with results investigating specific tension and atrophy, our results clearly imply that unloading-induced dysfunction of the NMJ can be implicated in the exacerbated negative effects of disuse on the juvenile neuromuscular system. Recall that relative loss of muscle mass evoked by unloading was similar in mature and still developing neuromuscular systems, so changes in muscle mass could not explain age specific unloading-induced decrements in peak force production. Providing further support that the NMJ is the key player in this unloading-related loss of strength is the fact that age-related difference in muscle mass of the soleus was present both at the start and finish of the testing protocol. In contrast, differences in peak tension were evident only at the onset of the stimulation protocol. Clearly, then, differences in muscle mass cannot explain age-specific unloading declines in force generating capacity. Moreover, electrical stimulation – a proxy for neural stimulation - to initiate muscle contraction was constant to both mature and juvenile muscles, erasing differences in pre-synaptic neural drive in explaining distinctions in strength production. The fact that differences in unloading elicited declines in strength are no longer apparent by the end of the stimulation regimen are consistent with previous literature suggesting that the NMJ is the “weak link” in fresh, but not fatigued muscle (Stephens and Taylor, 1972).

All told, results from this investigation confirmed our initial hypothesis driving this study. That is, due to the heightened plasticity naturally featured by the juvenile neuromuscular system as it progresses through its natural development, it is acutely sensitive to the well-known deleterious effects of muscle unloading. In particular, function of the NMJ among juvenile, but not mature, individuals is severely impaired by muscle unloading. At this point, it is unclear whether this synaptic disturbance is attributed more to pre- or post-synaptic factors, i.e., release, or binding of neurotransmitter, respectively, or even whether responsibility can be equally assigned to a pre- and post-synaptic origin. Additional research will be needed to better understand the hypersensitivity of the juvenile, rapidly developing neuromuscular system to unloading. Such information will have important, meaningful applications in physical medicine and rehabilitative efforts for the many juveniles who whether through injury, or illness, must undergo extended periods of neuromuscular disuse.

## Data Availability Statement

The raw data supporting the conclusion of this article will be made available by the authors, without undue reservation.

## Ethics Statement

The animal study was reviewed and approved by William & Mary Institutional Animal Care and Use Committee.

## Author Contributions

All authors listed have made a substantial, direct and intellectual contribution to the work, and approved it for publication.

## Conflict of Interest

The authors declare that the research was conducted in the absence of any commercial or financial relationships that could be construed as a potential conflict of interest.

## Publisher’s Note

All claims expressed in this article are solely those of the authors and do not necessarily represent those of their affiliated organizations, or those of the publisher, the editors and the reviewers. Any product that may be evaluated in this article, or claim that may be made by its manufacturer, is not guaranteed or endorsed by the publisher.
